# Runx Expression Is Mitogenic and Mutually Linked to Wnt Activity in Blastula-Stage Sea Urchin Embryos

**DOI:** 10.1371/journal.pone.0003770

**Published:** 2008-11-20

**Authors:** Anthony J. Robertson, Alison Coluccio, Peter Knowlton, Carrie Dickey-Sims, James A. Coffman

**Affiliations:** 1 Mount Desert Island Biological Laboratory, Salisbury Cove, Maine, United States of America; 2 Stowers Institute for Medical Research, Kansas City, Missouri, United States of America; Louisiana State University Health Sciences Center, Louisiana

## Abstract

**Background:**

The Runt homology domain (Runx) defines a metazoan family of sequence-specific transcriptional regulatory proteins that are critical for animal development and causally associated with a variety of mammalian cancers. The sea urchin Runx gene SpRunt-1 is expressed throughout the blastula stage embryo, and is required globally during embryogenesis for cell survival and differentiation.

**Methodology/Principal Findings:**

Depletion of SpRunt-1 by morpholino antisense-mediated knockdown causes a blastula stage deficit in cell proliferation, as shown by bromodeoxyuridine (BrdU) incorporation and direct cell counts. Reverse transcription coupled polymerase chain reaction (RT-PCR) studies show that the cell proliferation deficit is presaged by a deficit in the expression of several zygotic wnt genes, including wnt8, a key regulator of endomesoderm development. In addition, SpRunt-1-depleted blastulae underexpress cyclinD, an effector of mitogenic Wnt signaling. Blastula stage cell proliferation is also impeded by knockdown of either wnt8 or cyclinD. Chromatin immunoprecipitation (ChIP) indicates that Runx target sites within 5′ sequences flanking cyclinD, wnt6 and wnt8 are directly bound by SpRunt-1 protein at late blastula stage. Furthermore, experiments using a green fluorescent protein (GFP) reporter transgene show that the blastula-stage operation of a cis-regulatory module previously shown to be required for wnt8 expression (Minokawa et al., Dev. Biol. 288: 545–558, 2005) is dependent on its direct sequence-specific interaction with SpRunt-1. Finally, inhibitor studies and immunoblot analysis show that SpRunt-1 protein levels are negatively regulated by glycogen synthase kinase (GSK)-3.

**Conclusions/Significance:**

These results suggest that Runx expression and Wnt signaling are mutually linked in a feedback circuit that controls cell proliferation during development.

## Introduction

Multicellular development requires that the basic processes of cell growth and proliferation be subjugated to a higher level ontogenetic program. In animals this is achieved by way of genetic *cis*-regulatory systems through which the expression of cell cycle control genes is made contingent upon the spatiotemporally specified regulatory states of development. These states are established by the nuclear activities of sequence-specific transcriptional regulatory proteins, many of which are deployed in response to intercellular signaling systems. The developmental deployment of transcriptional regulatory proteins and cell signaling components is in turn controlled by a regulatory network encoded genomically by DNA sequence-specific *cis*-*trans* regulatory interactions [1]. Genetic mutations that short-circuit this regulatory network are commonly associated with cancer.

Runt domain (Runx) transcription factors are sequence-specific DNA binding proteins that are essential for the coordination of cell proliferation and differentiation during animal development [2], involving context-specific regulatory logic that remains to be elucidated. In vertebrates Runx genes are essential for hematopoiesis, skeletogeneis, and neurogenesis, and play critical roles in the development of gastrointestinal and epidermal epithelia [2]–[8]. They are also involved in cell cycle control [9] and causally associated with leukemia and other types of cancer, manifesting attributes of both oncogenes and tumor suppressors [10]–[16]. Depending on *cis*-regulatory sequence context, Runx proteins promote the assembly of protein-DNA complexes involved in either transcriptional activation or repression [17], [18]. This context-dependent functionality is mediated in part by heterodimerization with a non-DNA-binding partner, CBFβ, which enhances Runx DNA binding and half-life [19], [20]. However, Runx proteins are able to bind DNA as monomers and it was recently shown that CBFβ interacts with Runx facultatively rather than constitutively [21], suggesting that CBFβ may be a regulatory subunit that contributes to the context-dependency of Runx function.

Runx proteins contribute critically to the transduction of developmental signals via several key pathways, including those mediated by TGFβ/BMP, FGF, Notch, and Wnt proteins [22]–[32], each of which is essential for embryogenesis and stem cell regulation. Canonical Wnt signaling, which occurs through β-catenin bound to the HMG-box DNA binding protein Tcf/Lef, is required for stem cell self-renewal and progenitor cell proliferation in numerous vertebrate and invertebrate tissues, and de-regulation of this pathway is commonly associated with leukemia as well as epithelial cancers [33]–[36]. Canonical Wnt signaling stimulates growth and/or cell proliferation in part by activating the expression of D-type cyclins [34], [37], which drive cell cycle progression from G0 to G1 and from G1 to S phase in response to a variety of developmental signals. Since the sequence-specificity of Tcf/Lef is relatively low, it generally binds its target sites in cooperation with other transcription factors that bind near or adjacent the Tcf/Lef recognition sequence [24], [38]. Runx proteins have been shown in some *cis*-regulatory systems to be Tcf/Lef partners [24], and to thus facilitate the transduction of canonical Wnt signaling.

The genome of the sea urchin *Strongylocentrotus purpuratus* encodes two Runx genes [39], only one of which (*SpRunt-1*) is expressed during embryogenesis [40]. *SpRunt-1* is zygotically activated at late cleavage stage, and its pattern of expression in the embryo and larva is isomorphic with the pattern of growth and cell proliferation [40], [41]. Depletion of *SpRunt-1* mRNA and/or protein using morpholino antisense oligonucleotides (MASOs) leads to extensive gastrula-stage apoptosis and developmental arrest, which is attributable at least in part to the underexpression of the conventional protein kinase C *SpPKC1*, a direct SpRunt-1 regulatory target [42]. Here we extend our investigation of Runx function in sea urchin embryogenesis, showing that the earliest developmental defects associated with blockade of *SpRunt-1* expression include deficits in blastula stage cell proliferation and *wnt* gene expression. Furthermore, we find that SpRunt-1 protein levels are regulated by the activity of glycogen synthase kinase 3 (GSK-3), suggesting that Runx expression and canonical Wnt signaling are mutually linked.

## Results and Discussion

### SpRunt-1 expression is required for late blastula stage mitogenesis

**Figure 1:**
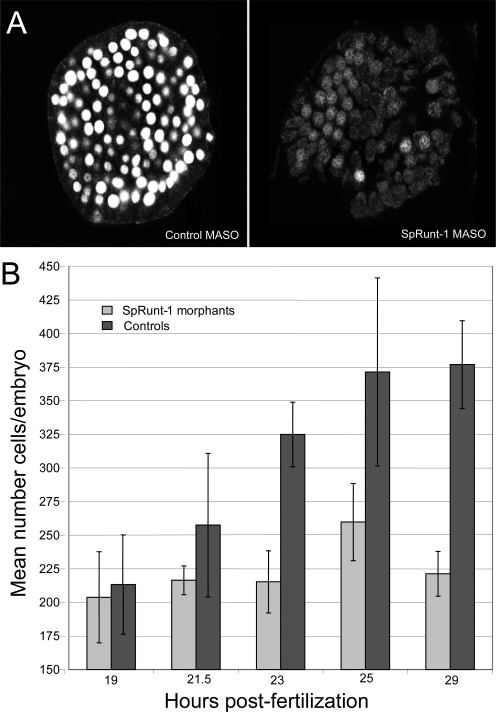
Blocking Runx expression causes a cell proliferation deficit in blastula stage sea urchin embryos. (A) Immunofluorescence labeling of BrdU incorporated from 18–24 hpf in control and SpRunt-1MASO-injected embryos. (B) Average cell numbers from four control-injected and four SpRunt-1 morphants at multiple time points from hatching to mesenchyme blastula stage. The error bars show the standard deviations.

Microinjection of zygotes with either a translation-blocking MASO that targets the 5′UTR near the translation start site or a splice-blocking MASO that targets the second exon-intron junction in the *SpRunt-1* transcript leads to development of blastulae that hatch on schedule and appear more or less normal, but which are somewhat smaller than their control-injected counterparts at mesenchyme blastula stage [21], [42], [43]. These embryos contain about half the DNA content of controls, and exhibit little or no apoptosis at this stage (data not shown). To ask whether cell cycle transit is defective in SpRunt-1 morphants at late blastula stage, embryos were pulse-labeled with bromodeoxyuridine (BrdU) from 18–24 hours post-fertilization (hpf), fixed, and stained with a fluorescent anti-BrdU antibody. Whereas control embryos display extensive nuclear BrdU incorporation throughout the embryo, SpRunt-1 morphants do not (Fig. 1A), indicating that SpRunt-1 expression supports progression of the cell cycle through S-phase in late blastula stage embryos.

To determine the precise temporal onset of the cell proliferation defect in SpRunt-1 morphants, embryos were labeled with a fluorescent DNA stain at different time points, squashed beneath cover slips to display the labeled nuclei in one plane, and fluorescently imaged [44]. Counts of labeled nuclei show that cell numbers are normal in the SpRunt-1 morphants up until 19–20 hours (hatched blastula stage), at which time both the morphant and control embryos contain ∼200 cells per embryo (Fig. 1B) [44]. However, between 20–24 hours the control embryos undergo an additional round of cell division, producing ∼400 cells per embryo, whereas the SpRunt-1 morphants do not (Fig. 1B) [44]. These data concur with the BrdU labeling results, and indicate that SpRunt-1 is required for continued mitogenesis in mesenchyme blastula stage embryos.

### SpRunt-1 supports mitogenic *wnt* and *cyclinD* expression

**Figure 2:**
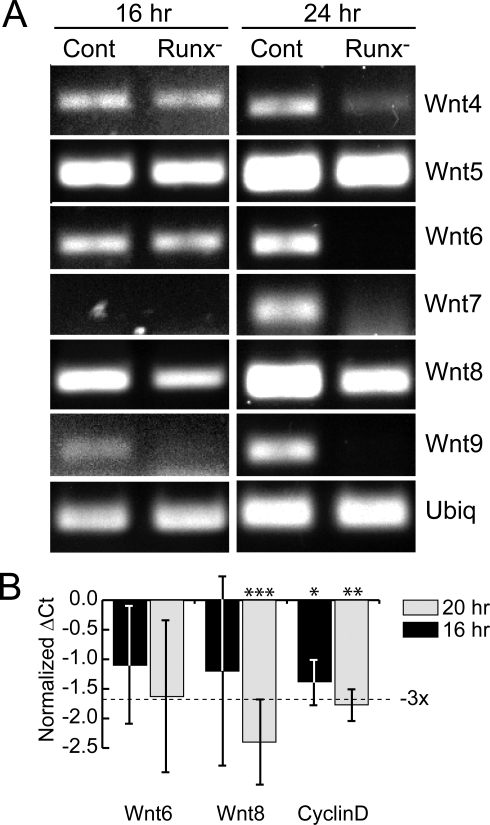
RT-PCR analysis of blastula stage *wnt* gene expression. (A) RT-PCR products obtained from control and SpRunt-1 morphants at 16 and 24 hpf using primer sets specific to several zygotically-expressed *wnt* genes, displayed by agarose gel electrophoresis. The intensity of the bands gives a rough indication of the relative levels of expression. The RT-PCR product for ubiquitin shows that approximately equivalent amounts of RNA were used in each sample. (B) Quantitative RT-PCR showing the ubiquitin-normalized difference in cycle number needed to achieve threshold fluorescence (ΔCt) in real-time RT-PCR of *wnt6*, *wnt8*, and *cyclinD* at 16 and 20 hpf. The ΔCt corresponding to a 3-fold difference in transcript abundance is indicated. Each bar represents the average of three or more separate measurements, except in the case of *wnt6*, which represents two measurements for the 16 hr sample. The number of biological replicates used to obtain each average was as follows: for *wnt6*, one at 16 hrs and two at 20 hrs (two and three measurements, respectively); for *wnt8*, two per time point (three measurements each); and for *cyclinD*, one per time point (three measurements each). The error bars show the standard deviations. Statistical significance calculated using a t-test is indicated by asterisks: *P = .0049; **P = .0005; ***P<.0001.

Canonical Wnt signaling is mitogenic in a variety of developmental contexts, and its transcriptional effector Tcf/Lef bound to β-catenin has been shown to interact with Runx proteins [24]. The sea urchin genome encodes 11 *wnt* genes, several of which are expressed at varying levels in the embryo [45]. We used RT-PCR to ask whether expression of any of the embryonically-expressed *wnt* genes is affected by knockdown of SpRunt-1 in the blastula stage embryo. Remarkably, the six *wnt* genes whose transcripts accumulate zygotically (*wnts* 4, 5, 6, 7, 8, and 9) were all found to be underexpressed in SpRunt-1 morphants, either prior to (16 hpf) or coincident with (24 hpf) the proliferation deficit observed at late blastula stage (Fig. 2A).

We chose to focus our attention on *wnt8*, as this appeared to be the *wnt* gene that was most affected by SpRunt-1 knockdown prior to the onset of the cell proliferation defect, and is to date the only sea urchin *wnt* gene that has been functionally characterized. *Wnt8* expression is localized to the presumptive endomesoderm and is required for specification of that territory [46]–[49]. Quantitative RT-PCR shows that *wnt8* is significantly (more than 4-fold) underexpressed in SpRunt-1 morphants at 20 hrs (Fig. 2B), the stage at which these embryos begin to manifest a cell proliferation defect. At this stage *wnt6* is expressed at much lower levels and is less strongly affected (Fig. 2B), although by 24 hrs *wnt6* is also significantly underexpressed (≥12-fold by QRT-PCR) in SpRunt-1 morphants, as are *wnt7* and *wnt9* (Fig. 2A). *CyclinD*, a mitogenic effector of canonical Wnt signaling which was shown previously to be positively regulated by SpRunt-1 at 48 hrs [43], is also significantly underexpressed at both 16 hrs (∼2.4 fold) and 20 hrs (∼3.5-fold) (Fig. 2B).

**Figure 3:**
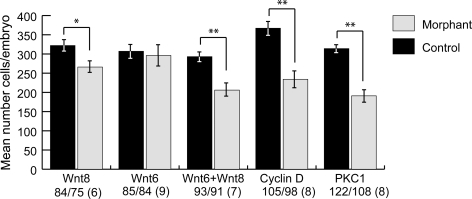
The effect of MASO-mediated knockdown of *wnt8*, *wnt6*, *wnt8*+*wnt6*, *cyclinD*, and *PKC1* on cell proliferation in blastula stage embryos. Each bar represents the average number of cells per embryo. The error bars show the standard errors of the mean. Significance was calculated using a *z*-test; *z>3, *P*<0.01, **z>4, *P*<0.001. The total number of embryos scored for each control/injected set is indicated under each heading on the x axis; the number of experimental repetitions for each set is in parenthesis.

*Wnt8* transcription is initially activated in the micromeres at the 16-cell stage, and its expression expands to the macromeres during subsequent cleavages, thereafter being extinguished in more vegetal cells such that by mesenchyme blastula stage *wnt8* activity is confined to a torus of presumptive endodermal cells [47], [49]. This is one of the regions of continued cell proliferation, which becomes confined to endomesoderm and oral ectoderm after mesenchyme blastula stage. To ask whether *wnt8* contributes to late blastula stage mitogenesis, we used a previously characterized MASO [49] to block translation of Wnt8 protein and examined the effect on cell numbers at 24 hrs. Blocking *wnt8* expression caused a modest but significant reduction in the number of cells per late blastula-stage embryo (Fig. 3). In contrast, Wnt6 knockdown did not have any effect on cell numbers at blastula stage (Fig. 3), although the MASO effectively depleted Wnt6 (Fig. S1) and did cause various morphological defects later in development (not shown). The fact that Wnt8 knockdown doesn't recapitulate the more extensive cell proliferation deficit displayed by SpRunt-1 morphants is probably attributable to the fact that *wnt8* is expressed in a more limited domain that contains only a subset of proliferating cells. In addition, it is possible that there is cross-regulation between *wnt* genes, which might produce compensatory effects on cell proliferation when expression of one or the other is knocked down. We therefore tested the effect of blocking expression of both Wnt8 and Wnt6 to more closely mimic the situation in SpRunt-1 morphants. Combined blockade of Wnt6+Wnt8 was found to produce a more significant cell number deficit than knockdown of Wnt8 alone (Fig. 3).

As noted above, Cyclin D is a key mitogenic effector of Wnt signaling. A previous report suggested that knockdown of Cyclin D does not affect cell numbers in sea urchin embryos [50]. However, in that study cells were only counted at late gastrula stage, and not blastula stage, leaving open the possibility that Cyclin D morphants manifest an early, transient deficit in cell proliferation that may later be compensated by regulative processes. We tested this possibility by counting cells in Cyclin D morphants at late blastula stage. As shown in Fig. 3, these embryos have about two-thirds as many cells as controls at 24 hrs, a deficit almost as large as that found in SpRunt-1 morphants. A similarly severe deficit in cell numbers was produced by knockdown of PKC1, consistent with the well-known mitogenic role played by this kinase, the gene for which was previously shown to be a Runx regulatory target [42]. Together, these results suggest that mitogenic function of SpRunt-1 is likely to be mediated by a complex battery of downstream regulatory targets including (but not limited to) *wnt8*, *cyclinD*, and *PKC1*, and hence not simply attributable to any single pathway or effector.

### SpRunt-1 binds sequences in the promoter regions of *cyclinD*, *wnt6* and *wnt8*, and is required for operation of a key *wnt8 cis*-regulatory module

**Figure 4:**
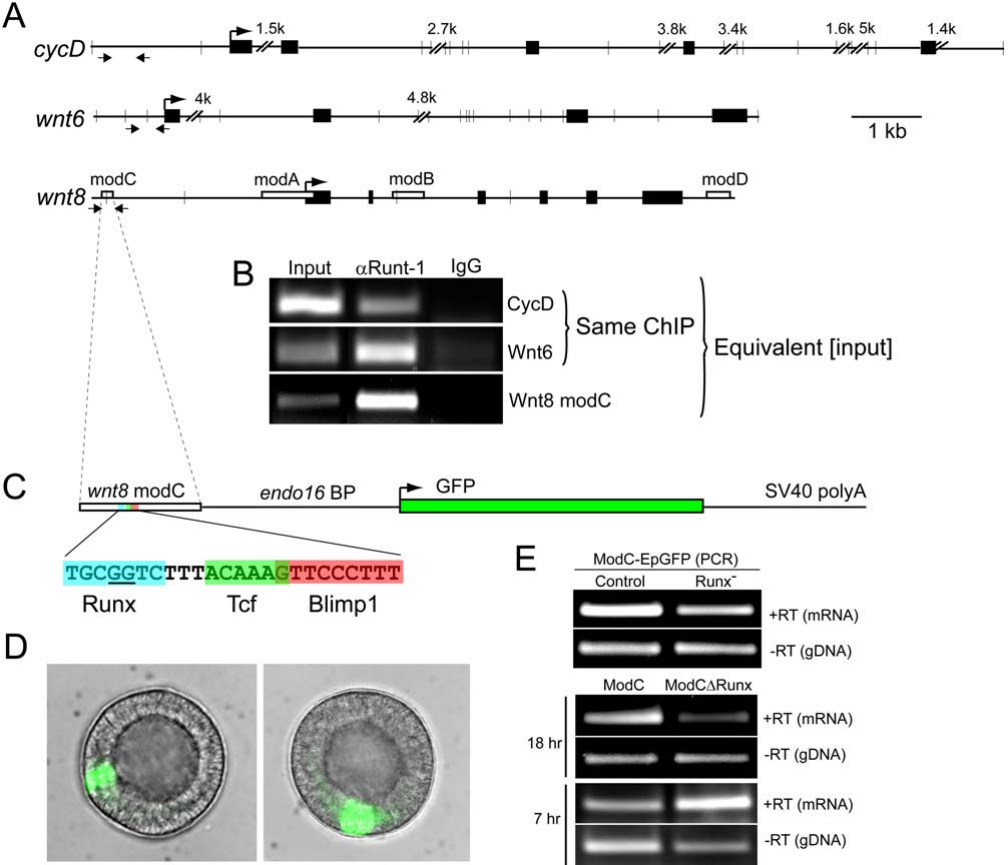
SpRunt-1 is bound to DNA in the 5′ flanking regions of *cyclinD*, *wnt6*, and *wnt8* in 20 hr blastula stage nuclei, and is required for blastula-stage activity of *wnt8* cis-regulatory module C. (A) Schematic representation of *cyclinD*, *wnt6*, and *wnt8*. Exons are shown as black bars. The previously-characterized *wnt8* cis-regulatory modules [48] are shown as open bars. Locations of the consensus Runx binding motif (TG^T^/_C_GGT) are indicated by vertical lines. Arrows show approximate primer locations for ChIP analysis. (B) PCR amplicons of *cyclinD*, *wnt6*, and *wnt8* obtained from ChIP of 20 hr embryo chromatin using anti-SpRunt-1 polyclonal IgG, or an equivalent quantity of non-immune IgG. In initial experiments, real-time PCR was used to determine a threshold number of cycles needed to obtain non-saturating signals from both the input DNA and SpRunt-1 ChIP DNA; this cycle number was then used as an end point in the experiments depicted here. Since an equivalent quantity of input DNA was used as template in each PCR, the relative band intensities give a rough indication of the enrichment obtained for each sequence. Thus, the *wnt6* amplicon (which centers on Runx target site) is shown to be substantially enriched by ChIP compared to the *cyclinD* amplicon (which does not center on a Runx target site). (C) Schematic of modC-EpGFP (not to scale). (D) Examples of modC-EpGFP expression in hatched blastulae. (E) RT-PCR analysis comparing modC-EpGFP expression in control and SpRunt-1 morphants, and to expression of modCΔRunx-EpGFP. The PCR products obtained without reverse transcriptase (RT) shows the relative levels of transgene incorporation for each experiment.

A survey of genomic sequence flanking the *cyclinD*, *wnt6*, and *wnt8* genes revealed numerous instances of the Runx consensus binding motif TG^T^/_C_GGT within upstream, intronic, and downstream regions (Fig. 4A). Sequences from the 5′ flanking regions of each of these genes were recovered by chromatin immunoprecipitation (ChIP) using a SpRunt-1-specific antibody, suggesting that SpRunt-1 binds DNA in the vicinity of these sequences in blastula stage embryos (Fig. 4B). Moreover, the ChIP enriched for sequences centered on a Runx binding site as compared to sequences displaced some distance from a Runx binding site (Fig. 4B; compare results from *cyclinD* and *wnt6*). These data indicate that Runx target sites in the 5′ flanking regions of *cyclinD*, *wnt6*, and *wnt8* are occupied by SpRunt-1 protein at late blastula stage.

Interestingly, one of the Runx binding sequences identified in *wnt8* occurs in a previously-characterized *cis*-regulatory module (‘module C’) that has binding sites for Tcf/Lef and Krox/Blimp1, the combination of which is necessary for β-catenin-dependent maintenance of *wnt8* activity [48]. Because Tcf/Lef is an HMG-box protein that binds the minor groove and bends DNA, thereby facilitating interactions between proteins bound at sites flanking either side of the Tcf/Lef site, Minokawa et al. [48] predicted that a third unidentified factor might bind immediately 5′ to the Tcf/Lef-Krox/Blimp1 sites in module C; this is precisely where the SpRunt-1 binding site is located (Fig. 4C). To test the functionality of this site, module C was cloned into a GFP *cis*-regulatory reporter construct (ModC-EpGFP) containing the naïve basal promoter from *endo16* [51] (Fig. 4C). It was shown previously that a module C-driven reporter gene with this promoter is expressed specifically in the endomesoderm precursors during cleavage stage, and globally at late blastula stage [48]. We verified that GFP is expressed in embryos developed from zygotes injected with ModC-EpGFP (Fig. 4D). RT-PCR shows that the level of this GFP expression is substantially reduced in blastula stage SpRunt-1 morphants (Fig. 4E) indicating that blastula stage activity of module C is dependent on SpRunt-1. Moreover, this dependency is due to direct interaction of SpRunt-1 with its target sequence in module C, as substitution of two base pairs essential for Runx binding to this sequence (Fig. 4C) abolishes blastula stage module C activity (ModCΔRunx; Fig. 4E). We conclude that at blastula stage, module C enhancer activity depends on sequence-specific interactions with SpRunt-1, which is likely to account at least in part for the blastula stage dependency of *wnt8* activity on SpRunt-1.

Zygotic activation of both *wnt8* and *runt-1* occurs at late cleavage stage (∼6 hpf), so it was of interest to examine whether the initial expression of *wnt8* is dependent on SpRunt-1. Since there is maternal SpRunt-1 protein (JAC, unpublished data), MASO-mediated knockdown might not be expected to affect *wnt8* expression, and this was found to be the case (data not shown). To address the question of whether early module C enhancer activity requires the Runx binding site, we compared the expression of ModC-EpGFP and ModCΔRunx-EpGFP in 7 hr (late cleavage stage) embryos. In contrast to the situation at 18 hrs, base substitutions that eliminate the Runx target sequence in module C do not abrogate module C-driven expression of GFP at this early stage; indeed, there appears to be an enhancement of expression (Fig. 4E).

These data provide further insight into the *wnt8 cis*-regulatory system. Initial expression of *wnt8* is confined to the micromeres at the 16–32 cell stage, and in subsequent development it expands outward to the macromere descendents in a dynamic torus [47]. This expression pattern is dependent on positive inputs from Blimp1 and Tcf complexed with vegetally localized β-catenin, the latter functioning to locally displace Groucho and thereby convert Tcf from a repressor into an activator [47]. These positive inputs are mediated in parallel by the *wnt8 cis*-regulatory modules A and C (Fig 4A) [48]. Our data suggest that during the early phase of *wnt8* expression, SpRunt-1 is dispensable for the positive enhancer activity of module C, and might even collaborate with Tcf/Groucho in repressing any non-specific or “leaky” module C activity (note that SpRunt-1, like other Runx proteins, has a Groucho recruitment domain at its C-terminus). By blastula stage however, module C enhancer activity becomes dependent on SpRunt-1, which is expressed throughout the embryo. This explains a previously unexplained observation: at blastula stage, module C-driven reporter gene expression occurs globally [48], whereas both Blimp1 and Tcf-β-catenin remain confined to the vegetal domain. This late non-localized activity of Module C can now be attributed to SpRunt-1, which explains the late spatial requirement for repressive intermodular interactions in the context of the *wnt8* cis-regulatory system [48]. The question of why module C becomes Runx-dependent later in development provides an interesting avenue for future research. One possibility is that this requirement is linked to structural constraints imposed on chromatin and/or nuclear architecture that occur in preparation for cell differentiation beginning at mesenchyme blastula stage.

### SpRunt-1 is negatively regulated by GSK-3

**Figure 5:**
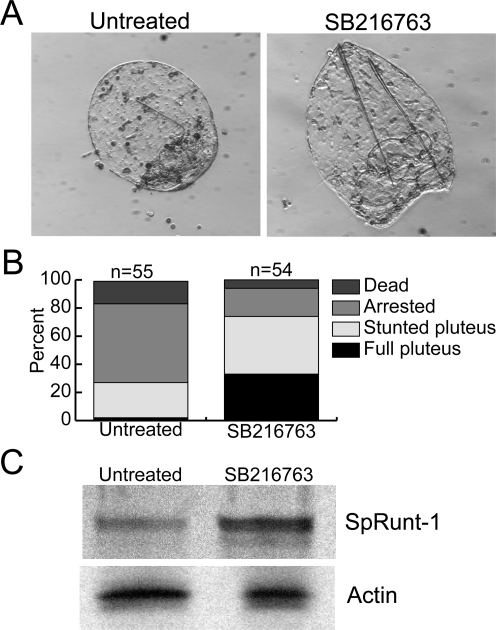
SpRunt-1 expression is negatively regulated by GSK-3. (A) Examples of SpRunt-1 morphants developed in the absence or presence of the GSK-3 inhibitor SB216763 beginning at blastula stage. The embryo on the left is an untreated three day old morphant; the one on the right is a three day old SB216763-treated morphant from the same group of injected embryos. (B) Quantitation of phenotypes obtained in the experiment shown in A. “Arrested” refers to a phenotype similar that on the left in A; “Full pluteus” refers to a phenotype similar to that on the right. “Stunted pluteus” refers to a phenotype intermediate between the two. (C) Immunoblot showing SpRunt-1 protein levels in equivalent numbers of normal blastulae and blastulae cultured from 20–24 hpf in the presence of SB216763. Actin serves as a loading control.

**Figure 6:**
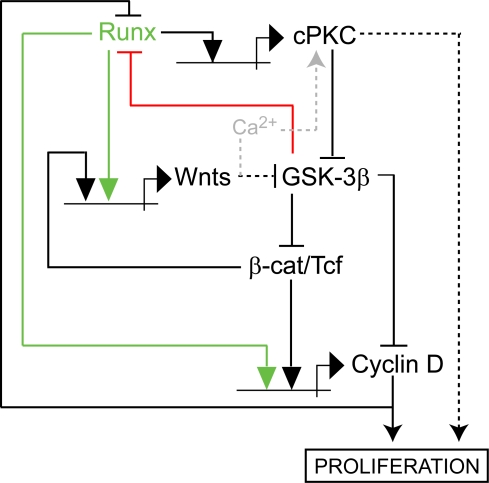
A hypothetical pan-metazoan regulatory circuit linking Runx expression to *wnt* activity and the developmental control of cell proliferation. Positive (activating) interactions are indicated by lines terminating in arrows; negative (inhibiting) interactions are indicated by lines terminating in bars. Both protein-protein and protein-DNA (*cis*-regulatory) interactions are shown; the latter are depicted by standard gene symbols (horizontal lines bearing a bent arrow). Interactions revealed in this work are shown in color; the others are gleaned from the literature (see text for supporting references).

To further explore the extent to which loss of *wnt* expression might contribute to the cell number deficit in SpRunt-1 morphants, we investigated the effect on cell proliferation of treating blastula stage morphants with GSK-3 inhibitors such as lithium or SB216763, which are expected to compensate for the loss of canonical Wnt signaling. Although lithium appeared in initial experiments to rescue cell numbers [44], SB216763 surprisingly rescued many other aspects of development in SpRunt-1 morphants: a substantial proportion of the inhibitor-treated morphants frequently developed into fully formed plutei (albeit with skeletal patterning defects), whereas their untreated cohorts underwent the typical developmental arrest associated with SpRunt-1 deficiency (Fig. 5A, B). Although one possible explanation for these results is that canonical Wnt signaling is the major effector of Runx function, another, more likely explanation stems from the fact that GSK-3β phosphorylates a number of transcription factors, including mitogenic factors such as Myc and Jun, and thereby targets them for destruction via the ubiquitin ligase fbw7 and the SCF complex [52]–[54]. We reasoned that SpRunt-1 levels may similarly be regulated by GSK-3β; and hence, that inhibition of GSK-3β may allow SpRunt-1 protein to accumulate to levels sufficient to overcome the MASO-mediated knockdown (note that the MASOs that we use only partially abrogate SpRunt-1 expression [43]). To ask whether this might be the case, we used immunoblot to compare SpRunt-1 protein levels in control and SB216763-treated blastula stage embryos. SpRunt-1 protein was found to be more abundant in the inhibitor-treated embryos (Fig. 5C), indicating that its steady-state levels are indeed negatively regulated by GSK-3. Although further studies are needed to determine whether this regulation is direct, involving GSK-3β-mediated phosphorylation of SpRunt-1, we note that the C-terminal sequence of SpRunt-1 has four serines and two threonines that are potential GSK-3 phosphorylation sites. Together with the fact that SpRunt-1 supports expression of multiple *wnt* genes, as well as expression of conventional PKC (which also antagonizes GSK-3 in some contexts [55], [56]), this result suggests that SpRunt-1 and GSK-3 are functionally antagonistic, and hence that Runx expression and canonical Wnt signaling are mutually linked in sea urchins (Fig. 6).

Runx proteins as well as components of the Wnt signaling pathway appear to be metazoan inventions, as they have not been found outside of the animal kingdom. Studies in nematodes [23] and vertebrates [24] have previously revealed functional cooperation between Runx proteins and Wnt signaling. Runx proteins and the Wnt signaling pathway are key regulators of animal stem cell proliferation in multiple contexts, and frequently associated with many kinds of cancer. For both Runx factors and the Wnt pathway, this mitogenic function is mediated in part by promoting the expression of D-type cyclins. Conversely, D-type cyclins have been shown to antagonize Runx protein function, both through direct physical interactions [57] and by promoting Runx protein degradation in collaboration with cdk4 [58]. Based on these observations and the results presented here, we propose that mutual linkages between Runx, Wnt, and Cyclin D activities constitute an ancient control circuitry (Fig. 6) that is a conserved module within the regulatory network that coordinates cell proliferation with patterning and differentiation in animal development.

## Materials and Methods

### Sea urchins, embryo culture, and microinjection

Sea urchins (*Strongylocentrotus purpuratus*) were obtained from Santa Barbara Marine Biologicals (Charles Hollahan, Santa Barbara, CA) or the Point Loma Marine Invertebrate Lab (Pat Leahy, Coronal del Mar, CA). Gametes were obtained by shaking. Eggs were fertilized with dilute sperm suspensions in artificial seawater (ASW), and embryos were cultured at 15°C in ASW. Microinjections were carried out using standard procedures [59].

### Morpholino antisense oligonucleotides and reporter gene constructs

Morpholino antisense oligonucleotides (MASOs) were obtained from GeneTools, LLC (Corvallis, OR). The translation blocking and splice blocking anti-SpRunt-1 (m2 and m5) and translation-blocking anti-SpPKC1 MASOs were described previously [42], [43]. The sequences of translation-blocking MASOs directed against SpWnt8 (GTACACTCCAATAAAAGAAATCAAA) and SpCyclinD (TATCCATGATTGATAGAAGACGTTC) were obtained from previous studies that established their efficacy [49], [50]. The sequence of a splice-blocking MASO directed against SpWnt6 was as follows: AAGACGTGAACTTACCACCAAAGAC; the efficacy of this MASO in knocking down Wnt6 mRNA was established by RT-PCR (Fig. S1). The standard non-specific control MASO from GeneTools was injected into control embryos at concentrations equivalent to those of the test MASO in all experiments.

### BrdU labeling and cell counts

Embryos were cultured in 300 µg/ml BrdU (Sigma-Aldrich) from 18–24 hours post-fertilization (hpf), then fixed in formaldehyde and prepared for confocal fluorescent imaging as described previously [42]. For cell counts, staged embryos were incubated for 60 minutes at 15°C in 50 µM Vybrant DyeCycle Green (Invitrogen Molecular Probes), a fluorescent stain for double-stranded DNA. The embryos were then gently squashed under cover slips to display all of the nuclei in one focal plane, and digitally imaged with a Zeiss Axiocam mounted on a Zeiss Axiovert microscope. The fluorescently-labeled nuclei were counted either manually, using transparencies mounted on the computer screen [44], or using NIH ImageJ software with the Cell Counter plug-in (http://rsb.info.nih.gov/ij/plugins/cell-counter.html).

### Quantitative reverse-transcription polymerase chain reaction (qRT-PCR)

Extraction of RNA from MASO-injected embryos, synthesis of random-primed cDNA, and qRT-PCR measurement (by SYBR-green fluorescence) of relative abundance of specific transcripts was carried out as previously described [21]. qRT-PCR measurements of threshold fluorescence (C_T_) were made using a SmartCycler (Cepheid), and ΔC_T_ between control and treatment embryos were normalized to the ΔC_T_ obtained for ubiquitin from the same samples. PCR products were analyzed by agarose gel electrophoresis to verify specificity of the products.

### Chromatin immunoprecipitation (ChIP) and *cis*-regulatory analysis

Chromatin immunoprecipitation from 20–24 hr blastula stage embryos using an anti-SpRunt-1 polyclonal IgG was carried out essentially as described [42] using a ratio of 2000 ng chromatin to 15 µg antibody and the final product was purified using the Qiaquick Nucleotide Removal Kit (Qiagen). DNA recovered by ChIP was analyzed by PCR using the cyclin D primers described previously [21], the *wnt8* module C primers described below, or the following primers for *wnt6*: CCTCTAGGTGGTAAAAAGATCCCCATCAA (forward) and ACCCTTCTCGCGGTTGCTGCAT (reverse).

The ModC-EpGFP reporter was constructed by cloning a restriction-digested PCR amplicon representing *wnt8* cis-regulatory module C [48] into the *KpnI* & *BglII* sites in the polylinker region of pEpGFP [51], which encodes GFP under the control of the *endo16* basal promoter. *Wnt8* module C was amplified from *S. purpuratus* genomic DNA using the following primers (restriction sites underlined): AAGGTACCTCCCAGCTCCCATTCTTACCCCGATT (forward) and ATGAGATCTGCCTGTCAGGTCCGGTAGGTATCTGAACAA (reverse). The QuickChange method (Stratagene) was used to substitute two residues critical for Runx binding within the Runx target site of ModC-EpGFP, using the following primers: GGCAGCCTCGCTATTGGTGCAATCTTTACAAAGTTCCC (forward) and GGGAACTTTGTAAAGAATGCACCAATAGCGAGGCTGCC (reverse). The resulting plasmids were linearized with *KpnI*. Linearized reporter plasmids (5 ng/µl) were co-injected with Sac1-digested sea urchin genomic carrier DNA (20 ng/µl), with or without anti-SpRunt-1 MASO m5 (600 µM).

To analyze reporter gene expression, total RNA was isolated from injected embryos harvested at blastula stage, and GFP mRNA was amplified by RT-PCR using the following GFP-specific primers: GAGCAAGGGCGAGGAACTGTTCACT (forward) and GCCATAGGTGAAGGTAGTGACCAGTGTT (reverse). The same primers were used to amplify residual genomic DNA in the RNA preparation (i.e., without reverse-transcriptase) to verify that control and treatment embryos had incorporated equivalent amounts of injected DNA.

### Inhibitor treatment and immunoblot analysis

Embryos were cultured in the presence 5 µM SB216763 (Tocris) or an equivalent amount of vehicle (DMSO) from 18–24 hpf, harvested, and extracted with ∼10 volumes of the total protein extraction reagent T-PER (Pierce). Following addition of ¼ volume of 4× LDS sample buffer containing β-mercaptoethanol, the samples were heated to 70°C for 15 minutes then subjected to SDS polyacrylamide gel electrophoresis on Novex MES gradient gels (Invitrogen). The contents of the gels were transferred to nitrocellulose, and subjected to immunoblot analysis using the Westernbreeze immunodetection kit (Invitrogen) and affinity-purified antibodies directed against the N-terminal peptide of SpRunt-1 [43] diluted to 2 µg/ml. An antibody directed against actin (Sigma) was used at a 1∶200 dilution as a loading control.

## Supporting Information

Figure S1 Efficacy of Wnt6 knockdown using a splice-blocking MASO. RT-PCR of Wnt6 from RNA extracted with embryos injected with a control MASO or a MASO that targets the first intron of Wnt6. Wnt1 is used as a specificity control.
(0.23 MB EPS)
